# Effect of Personalized AI‐Generated Picture Books on Anxiety During First Dental Visits in Young Children: Two Case Reports

**DOI:** 10.1155/crid/9947164

**Published:** 2026-05-26

**Authors:** Hitoshi Ishitani, Yuto Tanaka, Yoshiaki Ono

**Affiliations:** ^1^ Faculty of Dentistry, Osaka Dental University, Osaka, Japan, osaka-dent.ac.jp; ^2^ Department of Special Care Dentistry, Osaka Dental University Hospital, Osaka, Japan, osaka-dent.ac.jp

**Keywords:** artificial intelligence, behavior management, children, dental anxiety, picture book

## Abstract

This study tested whether a personalized picture book created using image‐generation technology reduces caregivers′ perceived child dental anxiety and improves cooperation during a child′s first dental visit. The book is read to achieve familiarity and predictability before the visit and is synchronized with the clinical sequence. Two dentally naive toddlers were recruited from a university clinic. For each child, a device‐based picture book was generated (using DALL·E 3) and tailored to the actual operation, attending dentist, and child′s appearance. Caregivers read the book at home (7 days previsit). The clinical sequence was intentionally managed to mirror situations depicted in the book ensuring continuity from home preparation. The sequence included sitting on a dental chair, introducing the instrument, oral examination, and topical fluoride application. Immediately after the visit, caregivers completed an online questionnaire (1–4 scales) assessing precaregiver/postcaregiver anxiety and worry about the child crying, perceived child cooperation, perceived contribution to anxiety reduction, intention to schedule periodic checkups, and perceived reading burden with free‐text feedback. Both caregivers reported lower anticipatory anxiety (pre‐reading mean: 3.5; postreading mean: 2.5) and worry about the child crying (pre‐reading mean: 4.0; postreading mean: 2.5). After the visit, they perceived the children to be highly cooperative (mean score: 4.0/4). Caregivers also felt that the picture book contributed to reducing their children′s anxiety (mean score: 3.0/4). For the second‐born child (single case), the caregiver reported lower anxiety than during the older sibling′s first dental visit (score: 4/4). Caregivers′ ratings of reading burden were mixed. Customized device‐based picture books that mirror real clinical settings and are synchronized with actual clinical visits appear feasible and acceptable for toddlers′ first dental visits. By offering familiar, concrete cues and a simple “what happens next” script, this approach may help mitigate negative expectations, support cooperation, and be adaptable to routine care.

## 1. Introduction

Early dental fear often persists in adolescence and adulthood, undermining routine attendance and treatment adherence throughout the course [1]. A recent systematic review and meta‐analysis estimated the pooled prevalence of dental fear and anxiety in 2–6‐year‐old children at approximately 30%, with children lacking prior dental experience showing significantly higher odds of experiencing dental fear [2]. Therefore, it is critical to ensure that a child′s first dental visit is positive and successful. Mechanistically, early negative experiences such as pain or invasive procedures promote fear acquisition, which is often reinforced by parental anxiety, negative information, and vicarious learning [3, 4]. In addition to these pathways, trait‐like dispositions, particularly sensory sensitivity and pain catastrophizing, are associated with higher dental anxiety and may increase susceptibility to fear consolidation [5]. Accordingly, interventions targeting these pathways at the earliest stage are preferred.

Given this risk profile, previsit preparation at home was used to foster familiarity and predictability before the first visit. Generic storybooks read by caregivers showed small to moderate reductions in anticipatory anxiety and modest gains in cooperation, supporting a low‐cost option for routine care [6, 7]. However, most evaluated materials were not personalized. Although tailored social stories are used for children on the autism spectrum, few rigorous studies isolate the added value of personalization [8–10]. Evidence is also limited for very young, dental‐naive children, and few approaches explicitly synchronize the preparatory content with the actual clinical experience [6–8]. These gaps highlight the need for interventions that remain low‐cost and acceptable to caregivers yet allow greater individualization and delivery synchronized with the clinical visit. The above strategies can be understood within the broader framework of anticipatory guidance and behavioral modeling, whereby providing children with predictable narratives about upcoming experiences facilitates emotional preparation and reduces threat appraisal [11]. From a theoretical standpoint, social learning theory posits that children acquire behavioral responses through observation of modeled actions; picture books depicting a calm, cooperative child protagonist may therefore serve as a form of symbolic modeling [3, 11]. Meanwhile, artificial intelligence (AI) is increasingly being explored in pediatric dentistry for applications including diagnostic imaging, caries detection, and behavior management [12]. However, the use of AI‐generated, personalized educational content for previsit preparation remains largely unexplored.

Therefore, this case report describes a pragmatic approach for creating customized visit‐synchronized picture books for caregivers using contemporary image generation tools. The books were tailored to the actual examination environment, attending dentist, and individual characteristics of the child. The clinical encounter was subsequently managed to intentionally mirror the prepared narrative, thereby maintaining continuity between the home preparation and the first dental visit.

Throughout this report, “caregiver” refers to the child′s parent or legal guardian.

## 2. Case Presentation

### 2.1. Setting and Participants

Two dental‐naive toddlers attended their first dental visit at a university dental clinic. Case A (20‐month‐old boy) was a firstborn child without older siblings. Case B (34‐month‐old girl) was a second‐born child with an older sister who had prior dental visits. Written informed consent for publication, including the use of de‐identified clinical information and AI‐generated images derived from children′s photographs, was obtained from their caregivers. Both children had unremarkable medical histories and were typically developing at the time of the study. Previous healthcare exposure was limited to routine pediatric visits (e.g., common colds) and standard vaccinations; their mothers reported no notable adverse reactions to prior medical encounters. Both caregivers reported experiencing dental anxiety themselves.

### 2.2. Intervention Workflow

#### 2.2.1. Picture‐Book Generation

**Figure 1:**
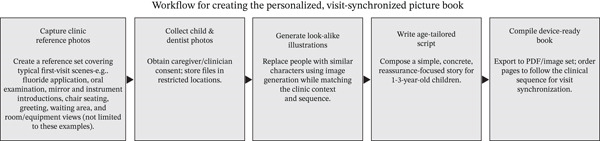
Workflow for creating the personalized, visit‐synchronized picture book: The clinic staff first captured reference photos of typical first‐visit scenes (fluoride application, oral examination, instrument introduction, chair seating, greeting, waiting area, and room/equipment views; examples not exhaustive). The caregivers and attending dentist then provided photographs with consent, which were stored in restricted locations and used solely as visual references to craft descriptive text prompts; no photographs were uploaded to external AI services. Using image‐generation tools, people in the references were replaced with look‐alike characters that matched the actual clinical context, and a short, reassurance‐focused story for 1–3‐year‐old children was written. The materials were compiled into a device‐ready PDF/image set and ordered to mirror the clinical sequence. Caregivers read the book at home about 7 days before the visit, the chairside course intentionally followed the same sequence, and the caregivers completed a brief postvisit questionnaire.

Using OpenAI′s image‐generation model (DALL·E 3), a picture book was created to depict the clinic approach, waiting room, greeting, sitting on the dental chair, dental mirror use, the air–water syringe, oral examination, topical fluoride application, and returning home. Visuals were matched to the real clinic layout/equipment and the dentist′s approximate appearance; the protagonist was modeled on a caregiver‐provided photograph of the child. Caregiver‐provided photographs were used solely as visual references by the investigators to craft descriptive text prompts; no photographs of the children were uploaded to external AI services. The text was simple and reassurance focused on using age‐appropriate language to facilitate toddler comprehension (Figure 1). The book comprised 10 pages, each featuring a single AI‐generated illustration and two to three short reassurance‐focused sentences (e.g., “Today we are going to the dentist. Let′s go see what it′s like!”). A total of 10 images were generated. Prompts were constructed around five categories: (1) clinic environment recreation (exterior, waiting room, and operatory); (2) character matching to approximate the child′s and dentist′s appearance; (3) instrument depiction (dental mirror and air–water syringe); (4) clinical procedure visualization (oral examination and fluoride application); and (5) emotional cues (calm expressions, curiosity, and reassurance).

#### 2.2.2. Home Reading (7 Days Before Visit)

Caregivers received the book as an image set (PDF) and were instructed to read it on a smartphone or tablet. The reading frequency was left to the caregiver′s discretion. Postvisit survey responses indicated that Case A′s caregiver read the book once or twice daily for approximately 5 min per session over the 7‐day period, whereas Case B′s caregiver read it four to five times in total, with each session lasting approximately 10 min.

#### 2.2.3. Visit Synchronization (Day of Visit)

To ensure continuity in home preparation, clinical experiences were intentionally managed to mirror the situations depicted in the picture book.

#### 2.2.4. Caregiver Survey

A Google Forms questionnaire was developed specifically for this exploratory case report and was not adapted from previously validated instruments. It captured eight items (1–4 scales: “completely negative” to “completely positive”) and a free‐text field. Caregivers completed the questionnaire once, immediately after the visit while still in the clinic. The items retrospectively captured pre‐reading, postreading/previsit, and postvisit perceptions within a single administration.

#### 2.2.5. Questionnaire Items

The items for the questionnaire are listed as follows: (i) From your perspective, does your child seem afraid of or reluctant to go to the dentist? (pre‐reading and postreading); (ii) worry that the child would cry during the visit (pre‐reading and postreading); (iii) After reading the picture book, how confident did you feel about bringing your child to the dental visit? (postreading); (iv) How cooperative would you say your child was during the dental visit? (postvisit); (v) (If applicable) If you have an older child, how did your child′s anxiety during this visit compare to your older child′s first dental visit? (postvisit); (vi) After this experience, do you intend to schedule periodic (regular) dental checkups for your child? (postvisit); (vii) Did you feel the picture book helped reduce your child′s anxiety about the dental visit? (postvisit); and (viii) Did you feel that reading the picture book with your child was burdensome (felt like a hassle or chore)? (postvisit), and a free‐text field for comments.

### 2.3. Feasibility and Completion

**Figure 2:**
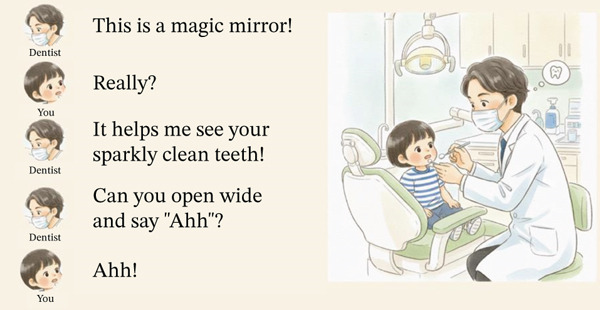
Example page from the personalized picture book: Sample spread used for at‐home preparation showing the dentist introducing the dental mirror to the child. The page presents short, reassuring dialogue (“This is a magic mirror… Can you open wide and say ‘Ahh’?”) that models the desired response. Illustrations match the actual clinic setting and the child′s appearance to enhance familiarity.

Both the toddlers attended their first dental visit as scheduled. Picture books were prepared and viewed at home on a device (smartphones or tablets). The intervention was completed successfully with the situations depicted in the book mirrored in an actual clinical setting. An example scene from the picture book is shown in Figure 2.

### 2.4. Chairside Course

In both cases, the children were seated directly on a dental chair. Clinical procedures, including the introduction of instruments, oral examinations, and topical fluoride application, were performed sequentially, as planned. Both children sat on the dental chair without resistance and remained calm throughout the visit. During the oral examination, neither child showed signs of distress or noncompliance. Introduction of instruments (dental mirror and air–water syringe) elicited curiosity rather than fear. Topical fluoride application proceeded without incident. Notably, neither child cried at any point during the visit.

### 2.5. Caregiver‐Reported Questionnaire

**Figure 3:**
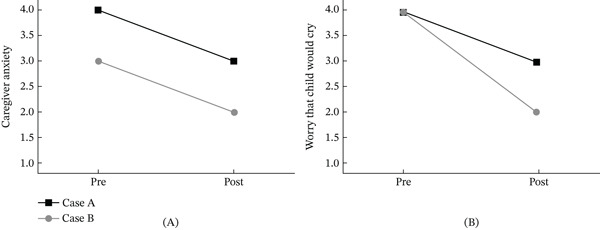
Pre–post changes after caregiver‐read, visit‐synchronized picture book: (A) caregiver‐perceived child dental anxiety. Line plot showing caregivers′ ratings of their child′s anticipated dental anxiety on a 4‐point scale (1 = *not at all*, 4 = *very much*). Each line represents a single Case A (square = Case A, circle = Case B). From pre‐reading to postreading of the personalized picture book, perceived child anxiety decreased from 4 to 3 for the boy and 3 to 2 for the girl, indicating lower anticipated anxiety before the first dental visit. Lower post values therefore reflect reduced caregiver‐perceived child dental anxiety; (B) caregiver worry that the child would cry. Line plot showing caregivers′ ratings on a 4‐point scale (1 = *not at all*, 4 = *very much*). Each line represents a single Case A (square = Case A, circle = Case B). From pre‐reading to postreading, worry decreased from 4 to 3 for the boy and 4 to 2 for the girl, indicating reduced anticipatory concern before the first dental visit. Lower post values reflected less worry and aligned with the broader pattern of reduced anxiety reported in the outcomes.

**Figure 4:**
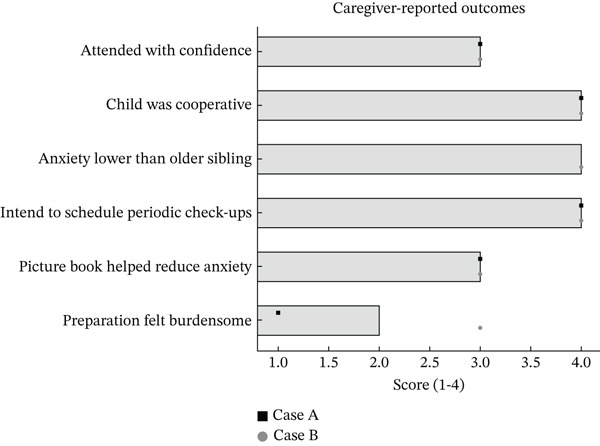
Caregiver‐reported outcomes: Horizontal bars show mean ratings on a 4‐point scale (1 = *not at all*, 4 = *very much*) across the two cases; overlaid symbols show individual scores (square = Case A, circle = Case B). Outcomes included the following: attended with confidence, child was cooperative, anxiety lower than older sibling (applicable only to the second‐born girl; value based on available case), intention to schedule periodic check‐ups, picture book helped reduce anxiety, and preparation felt burdensome (higher = greater burden). This pattern indicated high perceived cooperation and intention for follow‐up, moderate perceived contribution to anxiety reduction, and a low to moderate preparation burden.

Caregiver‐perceived child dental anxiety decreased from pre‐reading to postreading in both cases: Case A scored 4 pre‐reading and 3 postreading; Case B scored 3 pre‐reading and 2 postreading (mean change: 3.5–2.5) (Figure 3A). Worry that the child would cry also decreased: Case A from 4 to 3; Case B from 4 to 2 (mean change: 4.0–2.5) (Figure 3B). In the postvisit items, both caregivers reported attending with confidence (Case A: 3/4; Case B: 3/4; mean: 3.0/4) and perceived their child as cooperative (both scored 4/4; mean: 4.0/4). Both expressed an intent to schedule periodic checkups (both scored 4/4; mean: 4.0/4). Regarding the picture book′s contribution to reducing child anxiety, both caregivers rated it 3/4 (mean: 3.0/4). For the second‐born child (Case B), the caregiver reported lower anxiety than during the older sibling′s first dental visit (score: 4/4). Responses regarding reading burden were mixed; Case A′s caregiver rated it as low (1/4), whereas Case B′s caregiver rated it as moderately high (3/4) (detailed scores are shown in Figure 4).

### 2.6. Free‐Text Feedback

Caregivers noted that, while device‐based viewing was convenient, smartphone or tablet use sometimes prompted the child to touch the screen, suggesting that paper copies might avoid this. One caregiver also observed a positive collateral effect, noting that her child showed an improved attitude towards toothbrushing after the experience.

## 3. Discussion

This case report describes an intervention for dental‐naive children using personalized picture books created using image‐generation technology. Caregivers read these AI‐generated books to their children at home before their initial dental visit. A reduction in caregiver‐perceived dental anxiety was observed from before to after reading the picture book at home. This finding is of preventive significance as early attenuation of anticipatory anxiety may reduce the subsequent risk of dental phobia.

These picture books likely addressed several known pathways of dental fear. First, by depicting the actual room, dentist, and step‐by‐step flow with a protagonist modeled on the child, the book may have replaced or mitigated the negative preconceptions the child already held using concrete, clinic‐specific narratives and images [3]. Second, establishing continuity between the home preparation and the clinical visit probably created a simple “what happens next” script, which can enhance predictability and a sense of control [3]. This may be particularly relevant in children with higher sensory sensitivity [5]. Third, calm images and texts offer a nonthreatening model that can counter vicarious fear acquisition [3]. Although our data cannot confirm these mechanisms, the overall pattern is consistent with this explanation. These proposed mechanisms align with broader psychological theories of observational learning and emotional regulation in early childhood. According to social learning theory, children can acquire new behaviors and emotional responses by observing models in nonthreatening contexts [11]. In the present cases, the picture book protagonist—modeled on the child—demonstrated calm, cooperative behavior during each clinical step, potentially providing a template for behavioral imitation. Furthermore, the predictable narrative structure may have supported early emotional regulation by reducing the uncertainty that typically amplifies anxiety responses in young children.

Recent reviews support nonpharmacological behavioral interventions for pediatric dental anxiety, although the effect sizes vary according to the technique and procedure [13, 14]. Work in mobile health also indicates that delivering anxiety management content on common devices is feasible and acceptable for families [15]. This aligns with our device‐based approach and highlights practical considerations (e.g., tablet vs. paper) identified in caregiver feedback regarding toddlers who tend to touch screens. Beyond storybooks, broader digital adjunct toolkit that can complement tell–show–do when appropriate. For example, we previously demonstrated that virtual reality has shown promising effects on autonomic and subjective stress compared with nitrous oxide in a crossover study [16].

The use of AI image‐generation technology for creating personalized educational materials raises considerations regarding scalability, feasibility, and ethics. From a practical standpoint, the current workflow required approximately 30–60 min of investigator time per child to generate the picture book, suggesting that the approach could be integrated into routine previsit preparation with modest resource investment. However, the reliance on commercial AI platforms introduces variability in output quality and raises data privacy considerations, particularly when children′s photographs are used as reference material for image generation. In the present study, caregiver‐provided photographs were used solely as visual references by the investigators to craft descriptive text prompts; no photographs of the children were uploaded to external AI services. Future implementations should establish clear protocols for data handling, informed consent for AI‐based content creation, and transparent disclosure of AI use to caregivers [12].

### 3.1. Limitations

As the children were dental‐naive and first seen at the initial visit, we intentionally collected only a minimal, nonidentifying dataset to protect anonymity and feasibility; nonessential CARE items were marked as not applicable/not assessed with justification. This report has several notable limitations: the sample size (two cases); reliance on caregiver‐reported outcomes; the absence of objective behavioral or physiological measures; and no attempt to isolate the specific effects of personalization or digital delivery. Furthermore, caregiver ratings for reading burden were mixed, suggesting that the preparation phase may be demanding for some, warranting further investigation into the optimization of acceptability. Caregiver expectation bias cannot be excluded, as caregivers were aware that the intervention was designed to reduce their children′s anxiety, which may have influenced their subjective ratings. Additionally, the absence of a comparison group using standard (nonpersonalized) educational materials precludes determination of whether AI‐based personalization offers additional benefits beyond conventional approaches. Future research should involve pilot comparisons (generic vs. individualized; device vs. paper) incorporating validated behavioral scales and autonomic indices, examining children with high sensory sensitivity, and assessing attendance and adherence over time [1, 5, 13].

## 4. Conclusion

Customized, device‐based picture books that mirror the real clinical setting and are synchronized with actual clinical visits appear feasible and acceptable for toddlers′ first dental visits. Both children completed the visit without crying, and their caregivers reported reductions in anticipatory anxiety and high levels of child cooperation. By offering familiar, concrete cues and a simple “what happens next” script, this approach may help mitigate negative expectations and support cooperative behavior. Future research should include pilot comparisons of generic versus individualized materials, incorporate validated behavioral scales and objective physiologic measures, and evaluate whether personalized preparatory content confers measurable advantages over conventional approaches in larger samples.

## Author Contributions

**Hitoshi Ishitani:** conceptualization, investigation, writing – original draft. **Yuto Tanaka:** investigation, supervision, writing – review & editing. **Yoshiaki Ono:** supervision, writing – review & editing.

## Funding

This study was supported by the Osaka Dental University Student Research Grant.

## Ethics Statement

This case report was conducted in accordance with the Declaration of Helsinki. As a descriptive case report involving two participants from routine clinical care with no experimental allocation, formal institutional review board approval was not required under the institutional policy of Osaka Dental University. Written informed consent for participation and publication, including the use of de‐identified clinical information and AI‐generated images derived from children′s photographs, was obtained from the caregivers of both children.

## Conflicts of Interest

The authors declare no conflicts of interest.

## Data Availability

Data are available on request due to privacy/ethical restrictions.
